# Validation of the Davidson Trauma Scale in its original and a new shorter version in people exposed to the F-27 earthquake in Chile

**DOI:** 10.3402/ejpt.v4i0.21239

**Published:** 2013-08-22

**Authors:** Marcelo C. Leiva-Bianchi, Andrea C. Araneda

**Affiliations:** Faculty of Psychology, University of Talca, Talca, Chile

**Keywords:** Post-traumatic stress disorder, screening scale, exploratory factor analysis, confirmatory factor analysis, earthquake, tsunami, psychosocial impact

## Abstract

**Background:**

On February 27, 2010 (F-27), an earthquake and tsunami occurred having a significant impact on the mental health of the Chilean population, leading to an increase in cases of post-traumatic stress disorder (PTSD).

**Objectives:**

Within this context, validated for the first time in Chile was the Davidson Trauma Scale (DTS) using three samples (each one consisting of 200 participants), two of them random from the Chilean population.

**Results:**

Reliability analyses (i.e., *α*=0.933), concurrent validity (63% of the items are significantly correlated with the criteria variable “degree of damage to home”) and construct validity (i.e., CMIN = 3.754, RMSEA = 0.118, NFI = 0.808, CFI = 0.850 and PNFI = 0.689) indicate validity between regular and good for DTS. However, a new short version of the scale (DTS-SF) created using the items with heavier factor weights, presented better fits (CMIN = 2.170, RMSEA = 0.077, NFI = 0.935, CFI = 0.963, PNFI = 0.697).

**Discussion:**

Finally, the usefulness of DTS and DTS-SF is discussed, the latter being briefer, valid and having better psychometric characteristics.

In Chile, (Saturday, February 27, 2010 or F-27), an 8.8 Richter scale earthquake occurred, the sixth most powerful movement recorded since 1900 around the world (USGS, 2013). Later, a tsunami devastated several cities and towns along 300 km of central coast, between the cities of Constitucion and Talcahuano (PAHO, 2010). Moreover, almost 3 million people were heavily affected by F-27 in terms of damage to their cultural heritage, education systems, homes, and loss of human life (PAHO, 2010).

Based on the previous information, we can say that an earthquake and tsunami like that of F-27 are events that cause a very high impact on people, regardless of their ethnicity or socio-economic situation. Several studies have shown that F-27 had significant negative effects on the health of the inhabitants of central Chile (Figueroa, González, & Torres, 2010; Leiva, 2010; Leiva & Quintana, 2010; Mendez, Leiva, Bustos, Ramos, & Moyano-Díaz, 2010; MIDEPLAN, 2011; ONEMI, 2010). For instance, valid diagnostic tools and effective methods to quantify these effects are very important, especially in order to evaluate the most important mental health problem after a disaster: post-traumatic stress disorder (PTSD; APA, 2005; Rodríguez, Zaccarelli, & Pérez, 2006; Solvason, Ernst, & Roth, 2003).

Specifically, PTSD is an anxiety disorder that could be produced after exposure either directly or indirectly (hear stories, see pictures or movies) to extremely stressful and traumatic events (e.g., F-27). The traumatic event is re-experienced through rumination, uncontrollable and distressing memories or dreams, accompanied by images, thoughts, or perceptions. This produces an intense distress associated with continual avoidance of the experienced, dullness (reluctantly), behavioral activation and physiological responses. These responses appear especially when the person is exposed to internal or external cues that symbolize an aspect of the traumatic event. Some symptoms of PTSD are related to insomnia, inability to focus attention, irritability, anger, hyper-vigilance, and exaggerated startle response. These changes may last longer than 1 month and cause clinically significant distress or impairment in social, occupational, or other important areas of functioning (Lopez-Ibor & Valdes, 2008). Note that these symptoms do not always occur immediately after a disaster. The symptoms should appear at least 1 month after an event (APA, 2005). Some people affected by PTSD improve over time, while others may maintain the disorder for 4 years or more (Goenjian et al., 2000; Priebe et al., 2009).

Between 10 and 30% could be the prevalence of PTSD after disasters (Başoglu, Kılıç, Şalcıoglu, & Livanou, 2004; Başoğlu, Salcioğlu, & Livanou, 2005; Bland et al., 2005; Bulut, 2006; Cairo, Dutta, & Nawaz, 2010; Lai, Chang, Connor, Lee, & Davidson, 2004; McMillen, North, & Smith, 2000; Sharan, Chaudhary, Kavathekar, & Saxena, 1996). In Chile, after F-27, the prevalence of PTSD was 12%, 6% for men, and 15% for women (MIDEPLAN, 2011). Also, Leiva-Bianchi (2011) indicates that after 6 months, 36% of the inhabitants of Constitucion (a city significantly affected by the earthquake and tsunami), would be affected by post-disaster stress, a type of PTSD that includes symptoms of depression and altered daily functioning (Norris, Hamblen, Brown, & Schinka, 2008). Furthermore, it is expected that between 10 and 20% of health care personnel will have symptoms of PTSD and between 30 and 40% in camps of people that lost their homes after F-27 (Figueroa et al., 2010). Damage to the home, therefore, is a quite relevant variable in terms of event exposure. More symptoms of PTSD are observed in low-income groups because they experience a larger material impact (damage to their homes and loss of personal belongings) and because they have fewer resources with which to receive treatment (MIDEPLAN, 2011).

In regard to its measurement, there are a number of relatively short scales (screening) in order to perform a quick diagnosis of PTSD. One of the most common is the “Davidson Trauma Scale” (DTS). Davidson et al. (1997) proposed a scale composed of 17 items, each one related to DSM-IV symptoms. Regardless of the participants and their cultural characteristics, the DTS scale has both construct (generally in terms of three or four factors) and convergent validity (with other PTSD measurements), and very good internal consistency and test–retest reliability (Bobes et al., 2000; Chen, Lin, Tang, Shen, & Lu, 2001; Davidson et al., 1997; McDonald, Beckham, Morey, & Calhoun, 2009; Villafañe, Milanesio, Marcellino, & Amodei, 2003). For example, Villafañe and his collaborators (2003) indicate that DTS has very high reliability (*α*=0.890) and a structure composed of four factors very consistent with the original structure and other previous validity studies. It is probably for these reasons that DTS is widely used after potentially traumatic events.

However, DTS has some problems. Each item requires two answers, which could cause confusion and fatigue in people who suffer from PTSD. As mentioned, each participant responds twice to the 17 items that make up the scale, one answer evaluating frequency and the other evaluating intensity. Although this has not caused problems in scale validity (Chen et al., 2001), it could be a practical problem when applying DTS to people who have experienced potentially traumatic events and who are not emotionally prepared to respond to very long instruments. Another problem is that in a Chilean context, DTS has not been validated, although it has been applied on at least two occasions, both times after F-27: 3 months later as part of the Post Earthquake Questionnaire applied together with the National Socio-Economic Characterization Questionnaire (Encuesta de Caracterizacion Socioeconomica Nacional—CASEN) of the Social Development Ministry (MIDEPLAN, 2011); and 7 months later as a measurement criterion in order to validate the post-disaster stress scale (SPRINT-E; Leiva-Bianchi & Gallardo, 2013). In neither of the two cases were DTS reliability or validity indictors reported. In order to validate the DTS for the first time in Chile and taking data from both studies, we conducted this research.

## Method

### Sample and procedure

We selected three random samples of 200 participants each, belonging to two different databases. We chose 200 participants per sample given that it is the maximum limit recommended with which to carry out scale validation (Barret, 2007; Hair, Anderson, Tatham, & Black, 2004). Participants of the first and second samples belong to two regions particularly affected by F-27 (21 and 20% belong to the Metropolitan Region, and 79 and 80% to the Maule Region in both samples, respectively). The samples were selected randomly from the 2010 CASEN Post Earthquake Questionnaire database. Said survey is unique in that it was selected from a representative subsample of households interviewed. The selection was performed via a random sample stratified by sections and carried out in two phases. The sample was based on close to 27,000 participants (interviewed directly) throughout the country. Such a size provides for a margin of error of no more than 8% in all regions and provinces affected by the earthquake and avoids the inconvenience of no answer (MIDEPLAN, 2011). Such rigorousness in sampling is not common in scale validation studies, and even less so in samples of people affected by the same stressful and potentially traumatic event.

The third sample corresponds to 200 participants from the Metropolitan (21%) and Maule (79%) Regions. This time, it was a non-probability convenience sampling. Similar to the two previous samples, we were interested in groups of people belonging to regions affected by F-27, although in different degrees. These people were interviewed 7 months after the earthquake occurred.

### Instruments

#### Davidson Trauma Scale

The DTS validated in an Argentinean population was used having a good reliability index and good construct validity (Villafañe et al., 2003). Items are classified according to DSM-IV criteria for PTSD diagnosis: Criteria B “re-experimentation” (RE; items 1–5); Criteria C “avoidance and numbing” (AN; items 6–12); Criteria D “hyper-activation” (HA; items 13–17). For each item, the person performs two evaluations, both on a scale of 0 (never/nothing) to 4 (daily/extreme) points: one for frequency (number of times it has happened) and the other for intensity (magnitude or gravity) of the symptom experienced. The least possible score is 0, and the maximum is 136. A score of over 40 points is considered to indicate a high probability that the person suffers from PTSD (Davidson et al., 1997).

#### Degree of damage to home

In order to determine the degree of damage to the home of each participant, we used the question “As a result of the earthquake, what damage did your home incur?” We evaluated the degree of damage using the following levels: “no damage” (0), “light damage such as cracking” (1), “heavy damage such as fallen walls or ceilings” (2), and “total loss” (3).

### Data analysis

Before starting the validation process itself, the reliability of the instrument was tested by Cronbach's alpha (*α*) for the 17 items of the DTS. For this test, a value above 0.9 is considered excellent (Pardo & San Martin, 1998). In order to perform the reliability analysis, we used each of the three previously described samples separately.

Then, to assess construct validity, we performed an exploratory factor analysis (EFA) with a generalized least squares extraction method, free numbers of factors (criterion of eigenvalues greater than 1) and varimax rotation. Although we expect related factors, varimax rotation was performed because it is a simple way to interpret and we will carry out a confirmatory factor analysis (CFA), which is more accurate with the estimation of the relation between the factors. The EFA was performed using 200 randomly selected participants from the original sample of 26,737. The model has a good fit and it is relevant to perform the analysis if the following tests show values within the limits: Kaisser–Meyer–Olkin (KMO) > 0.51; Bartlett's sphericity test with *p*<0.01, *X*^2^ with *p*>0.05 (Ximenez & San Martin, 2004).

To confirm the existence of the pattern obtained from the EFA, we conducted a CFA through a structural equation model with the 17 items of DTS. CFA was performed using other samples of participants that were not used in EFA (*n*=200). Considering the arguments of Barret (2007), in this case a model has an appropriate fit if the following indicators have values approximately within the limits: CMIN/DF < 3, RMSEA < 0.05, TLI > 0.9, CFI > 0.9 and PNFI > 0.5 (Hair et al., 2004; Schreiber, Nora, Stage, Barlow, & King, 2006).

Once the single model is obtained, it is once again submitted to CFA, this time with a third sample: 200 participants selected via a non-probabilistic sampling, evaluated 6 months after F-27. The model fit will be evaluated according to the same indicators mentioned above.

Finally, to determine concurrent validity, Pearson correlations were conducted between DTS items with home damage level. The more the DTS items are related with home damage, the better the criterion validity will be. All these correlations must be statistically significant (*p*<0.05).

Both the analysis of reliability and EFA was performed using SPSS version 15. CFA was performed using AMOS version 16.

## Results

### Reliability and concurrent validity of original DTS

Regarding the reliability of DTS, the Cronbach's alpha values are 0.948, 0.933, and 0.942 for 17 DTS items in three different samples. Furthermore, when analyzing the values of this test, if any item is deleted, the Cronbach's alpha decreases. Not only that, in the three samples analyzed, the dimensions RE (0.873, 0.867, and 0.867), AN (0.870, 0.853, and 0.837), and HA (0.905, 0.891 and 0.913) showed very high reliability indexes. Again, analyzing the test values when any item is deleted, the alpha decreases.

As for concurrent validity, Table 1 shows the Pearson correlations between DTS and home damage item, indicating that there are 94, 41, and 53% of correlated items (*p*<0.01) in the three samples, respectively. There are also 11 items that are correlated with the criteria in at least two of the three samples. In total, 63% of items correlate with the criteria question.

**Table 1:** Significant relations between 17 DTS items and home damage item in three samples

	Home damage item			
	
DTS items	Sample 1	Sample 2	Sample 3	% sig
1RE	***0.234***	***0.258***	***0.267***	100
2RE	***0.281***	0.133	***0.233***	67
3RE	***0.182***	0.034	0.098	33
4RE	***0.154***	***0.291***	0.107	67
5RE	***0.163***	***0.156***	0.060	67
6AN	***0.181***	0.108	***0.226***	67
7AN	***0.249***	0.102	−0.002	33
8AN	***0.200***	***0.220***	***0.188***	100
9AN	***0.185***	−0.002	0.140	33
10AN	***0.210***	0.092	0.096	33
11AN	***0.150***	0.081	0.073	33
12HA	***0.245***	***0.177***	***0.192***	100
13HA	***0.156***	0.120	***0.233***	67
14HA	***0.218***	0.067	***0.194***	67
15HA	0.099	***0.189***	***0.170***	67
16HA	***0.176***	***0.181***	***0.150***	100
17HA	***0.169***	0.053	0.123	33
% Sig.	94	41	53	**63**

Note: All significant correlations (at least *p*<0.05) are in bold. In italics are significant at 5% (*p*<0.05), in italics and underlined, significant at 1% (*p*<0.01).

### Construct validity: *EFA*

In the first sample (*n*=200), EFA was performed to begin the DTS construct validity analysis. First, we analyzed the relevance of factor solution and if there is a structure of relations among the items suitable for extracting factors. In this regard, the KMO (0.915 and Bartlett's sphericity (*X*^2^=2600.963, *p*<0.01) tests indicated that the structure of correlations was adequate.

The factor structure of this solution was analyzed. In this regard, we obtained a three-factor solution that explained 63% of the total variance. This structure is similar to that found by other authors (Chen et al., 2001; McDonald et al., 2009; Villafañe et al., 2003) and that which was originally proposed (Davidson et al., 1997). However, upon examining the rotated component matrix, we found that Items six and seven originally belonging to the dimension AN, now weighed much more in RE. Something similar occurs with Item 13 of HA, which weighed more in RE (see Table 2). This, in light of the fact that these items could have a certain semantic coherence that would link them with the RE factor, motivates us to propose two models in order to confirm: one using the EFA (empirical) and the other using the original DTS dimensions (original).

**Table 2:** Rotated factor matrix for two factor solutions

		Factors		
		
Label	Item	1	2	3
1RE	“He tenido alguna vez imágenes, recuerdos o pensamientos dolorosos del acontecimiento”	**0.699**	0.306	0.265
2RE	“He tenido alguna vez pesadillas sobre el acontecimiento”	**0.636**	0.301	0.248
3RE	“He sentido que el acontecimiento estaba ocurriendo de nuevo, como si lo estuviera reviviendo”	**0.670**	0.315	0.309
4RE	“Hay cosas que me lo han hecho recordar”	**0.690**	0.128	0.287
5RE	“He tenido sensaciones físicas por recuerdos del acontecimiento (como transpiración, temblores, palpitaciones, mareos, náuseas o diarrea)”	**0.609**	0.241	0.361
6AN	“He estado evitando pensamientos o sentimientos sobre el acontecimiento”	**0.725**	0.322	0.177
7AN	“He estado evitando hacer cosas o estar en situaciones que me recordaran el acontecimiento”	**0.649**	0.420	0.179
8AN	“He sido incapaz de recordar partes importantes del acontecimiento”	0.342	0.222	**0.435**
9AN	“He tenido dificultad para disfrutar de las cosas”	0.480	0.378	**0.546**
10AN	“Me he sentido distante o alejado de la gente”	0.239	0.119	**0.752**
11AN	“He sido incapaz de tener sentimientos de tristeza o de afecto”	0.177	0.261	**0.689**
12AN	“He tenido dificultad para imaginar una vida larga y cumplir mis objetivos”	0.345	0.325	**0.666**
13HA	“He tenido dificultad para iniciar o mantener el sueño”	**0.576**	0.416	0.361
14HA	“He estado irritable o he tenido accesos de ira”	0.196	**0.627**	0.266
15HA	“He tenido dificultades para concentrarme”	0.410	**0.607**	0.317
16HA	“Me he sentido nervioso, fácilmente distraído, o como en guardia”	0.368	**0.828**	0.260
17HA	“He estado nervioso o me he asustado fácilmente”	0.388	**0.822**	0.213

Note: Factorial loadings in bold letter where item belongs to a factor. The original DTS items (Davidson et al., 1997) are: (1RE) “Have you had painful images, memories or thoughts of the event?”; (2RE) “Have you had distressing dreams of the event?”; (3RE) “Have you felt as though the event was re-occurring?”; (4RE) “Have you been upset by something which reminded you of the event?”; (5RE) “Have you been physically upset by reminders of the event?”; (6AN) “Have you been avoiding any thoughts or feelings about the event?”; (7AN) “Have you been avoiding doing things or going into situations which remind you about the event?”; (8AN) “Have you found yourself unable to recall important parts of the event?”; (9AN) “Have you have difficulty enjoying things?”; (10AN) “Have you felt distant or cut off from other people?”; (11AN) “Have you been unable to have sad or loving feelings?”; (12AN) “Have you found it hard to imagine having a long lifespan fulfilling your goals?”; (13HA) “Have you had trouble falling asleep or staying asleep?”; (14HA) “Have you been irritable or had outburst of anger?”; (15HA) “Have you had difficulty concentrating?”; (16HA) “Have you felt on edge, been easily distracted, or had to stay ‘on guard’?”; (17HA) “Have you been jumpy or easily startled?.”

### Construct validity: CFA

**Fig. 1:**
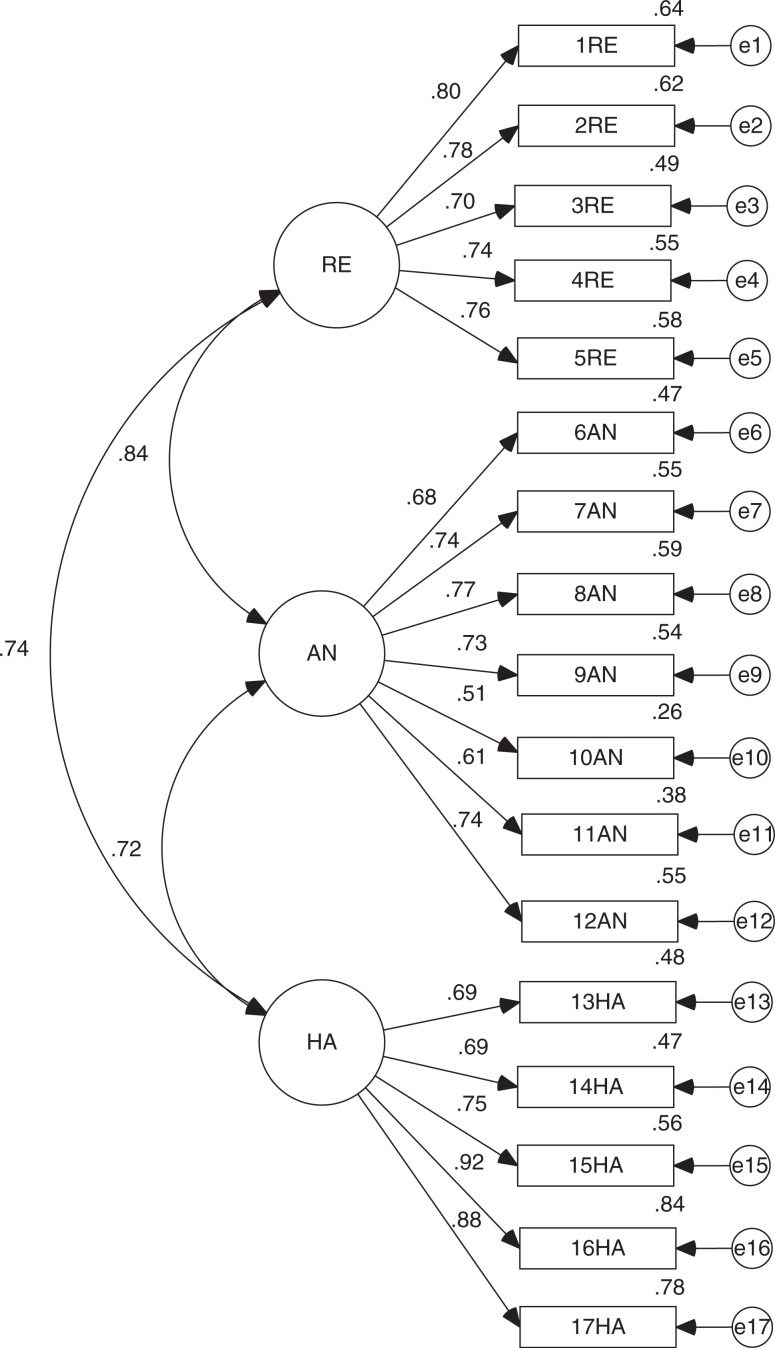
Standardized version of CFA for DTS original model.

Given the above results, we conducted a CFA to specify the fit of the empirical model and compare its fit with the original. In both cases, maximum likelihood estimation method was used. The analysis was performed on the second sample (*n*=200). To begin, in the original model, all factor loadings were significant (*p*<0.001). However, it has a regular overall fit (CMIN/DF = 3.754 and RMSEA = 0.118) and incremental fit (TLI = 0.824 and CFI = 0.850), although it provided a good fit of parsimony (PNFI = 0.689; see Fig. 1).

**Fig. 2:**
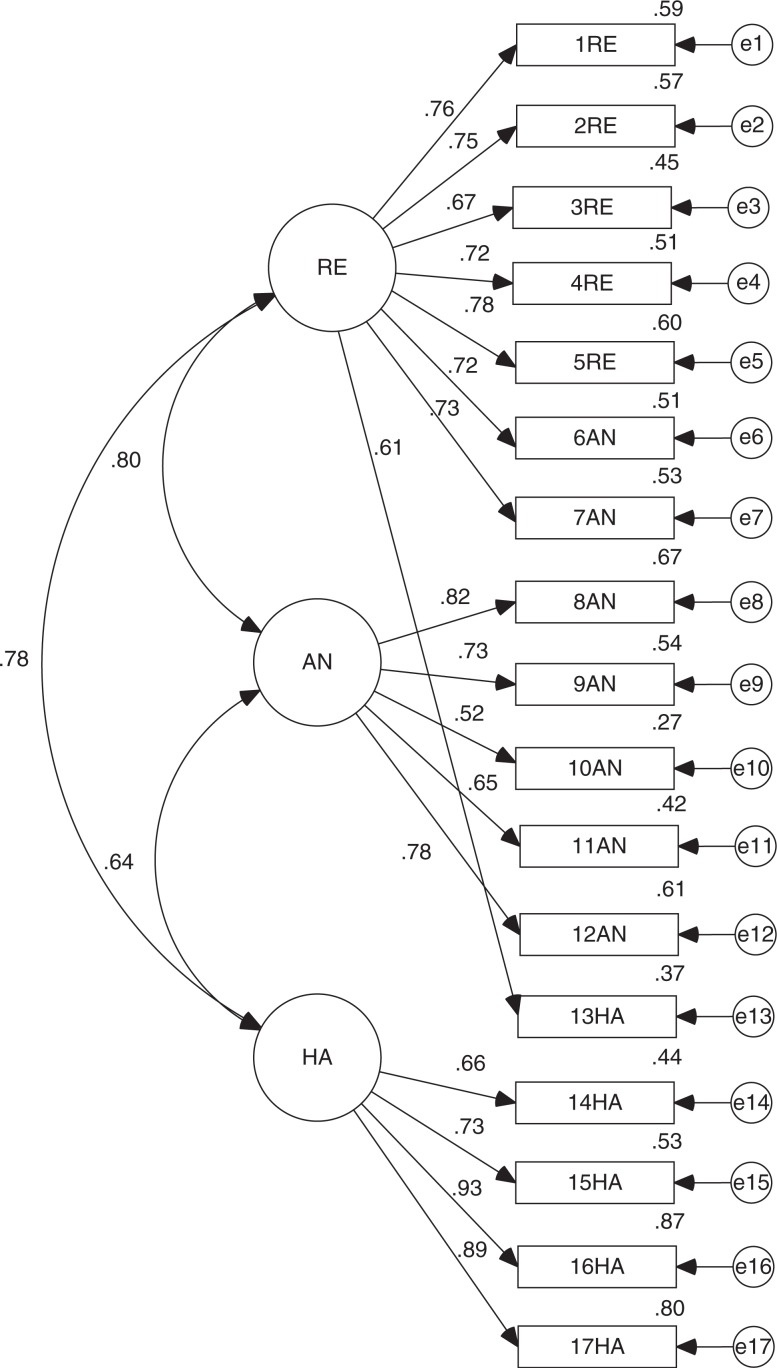
Standardized version of CFA for DTS empirical model.

Regarding the empirical model, all factor loadings were significant to (*p*<0.001). This model also had a regular general fit (CMIN/DF = 3.844 and RMSEA = 0.120) and incremental fit (TLI = 0.808 and CFI = 0.845). However, parsimony fit (PNFI = 0.685) shows an appropriate fit (see Fig. 2).

With the previous results, we can establish that both models showed the same fit. Given this problem, we opted for the model originally proposed for the DTS scale. For greater assurance, we carried out a CFA, adjusting the original and empirical model with a third sample (*n*=200). The results show the same trend found in the two previous models: all factor loadings were significant to (*p*<0.001), regular general (CMIN/DF = 3.230 and RMSEA = 0.106 for empirical and CMIN/DF = 3.271 and RMSEA = 0.107 for original model) and incremental fit (TLI = 0.866 and CFI = 0.886 for empirical and TLI = 0.863 and CFI = 0.883 for original model), and good parsimony fit (PNFI = 0.720 for empirical and PNFI = 0.718 for original model).

**Fig. 3:**
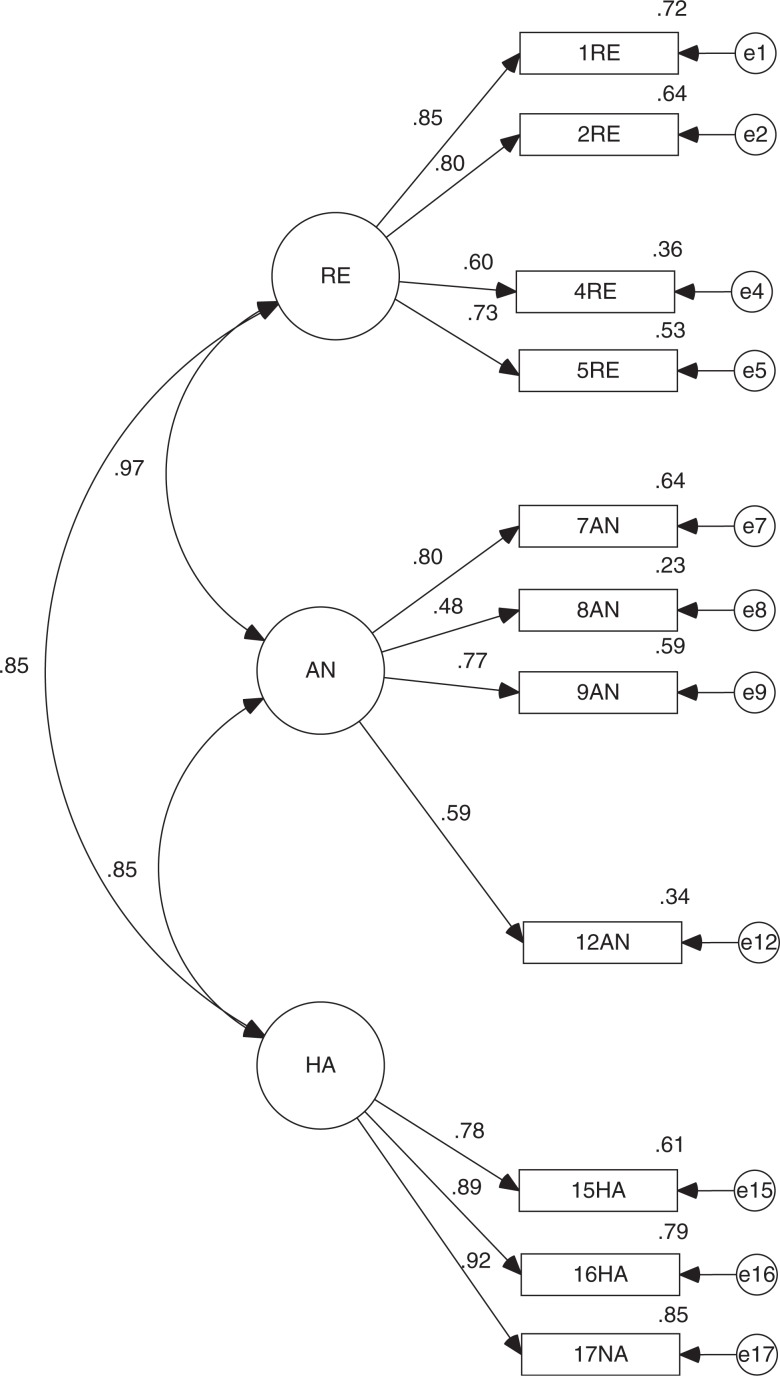
Standardized version of CFA for new DTS-SF.

However, we cannot ignore that the fit of the theory model appeared regular. Therefore, we designed a fourth model using that which was originally proposed, without items 3, 6, 10, 11, 13, and 14 for having the model's lowest weights (*λ*<0.7). This is how we arrived at a model that contains 11 items. By testing the model's fit with Sample 2, we obtained a regular general fit (CMIN/DF = 3.567 and RMSEA = 0.114), although the incremental (TLI = 0.896 and CFI = 0.923) and parsimony fit (PNFI = 0.669) are valid. Now, the fits based on Sample 3 are the best and bring us to the conclusion that the general (CMIN/DF = 2.170 and RMSEA = 0.077), incremental (TLI = 0.951 and CFI = 0.963) and parsimony fits (PNFI = 0.697) are valid (Fig. 3).

### Reliability of 11-item DTS version

Regarding the reliability of 11 items that DTS finally obtained, the Cronbach's alpha values of 0.925, 0.917, and 0.930 in three different samples, are excellent. Furthermore, when analyzing the values of this test if any item is deleted, the Cronbach's alpha decreases. Also, in the three samples analyzed, the dimensions RE (0.847, 0.847, and 0.824), AN (0.795, 0.823, and 0.756) and HA (0.851, 0.878, and 0.896) have very high reliability indexes. Again, analyzing the test values when any item is deleted, the alpha decreases.

## Discussion

**Fig. 4:**
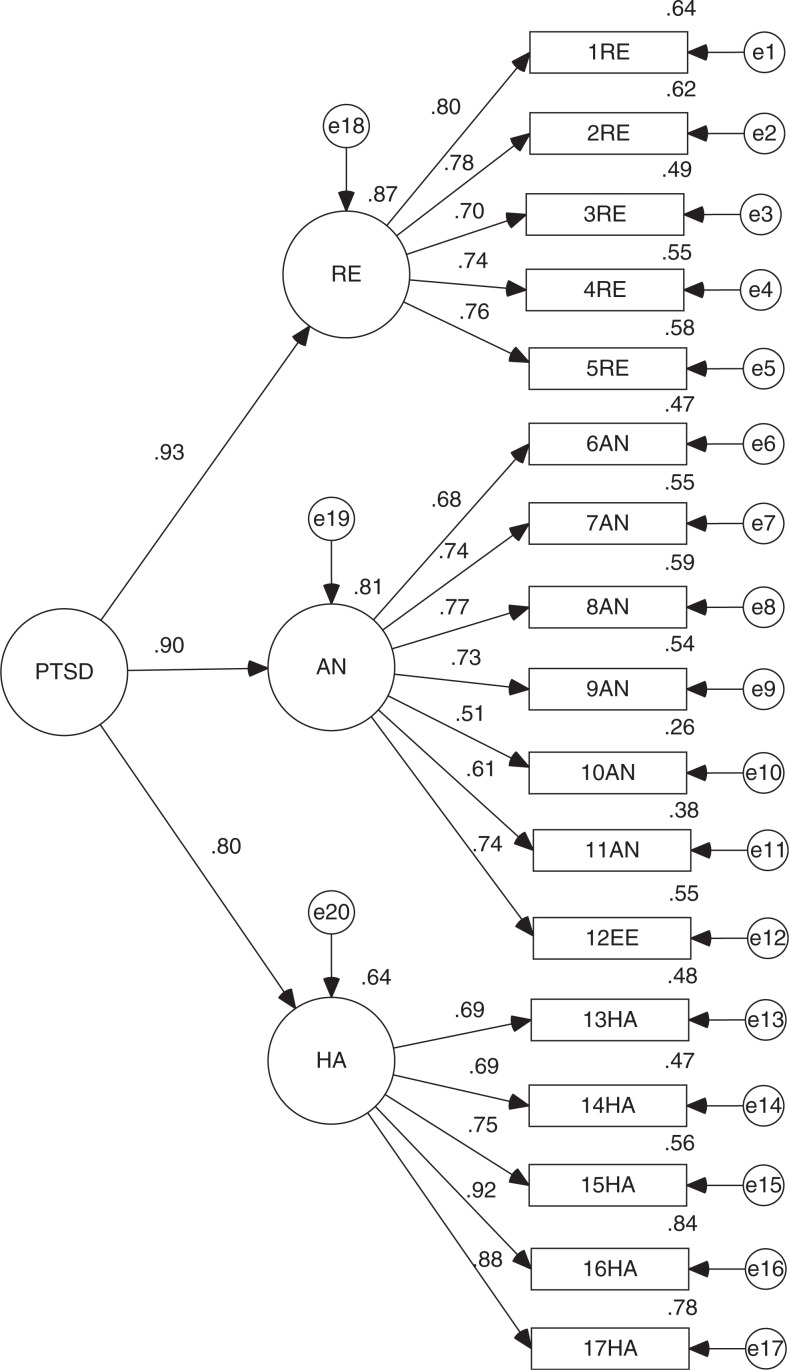
Standardized version of CFA for DTS second order model.

**Fig. 5:**
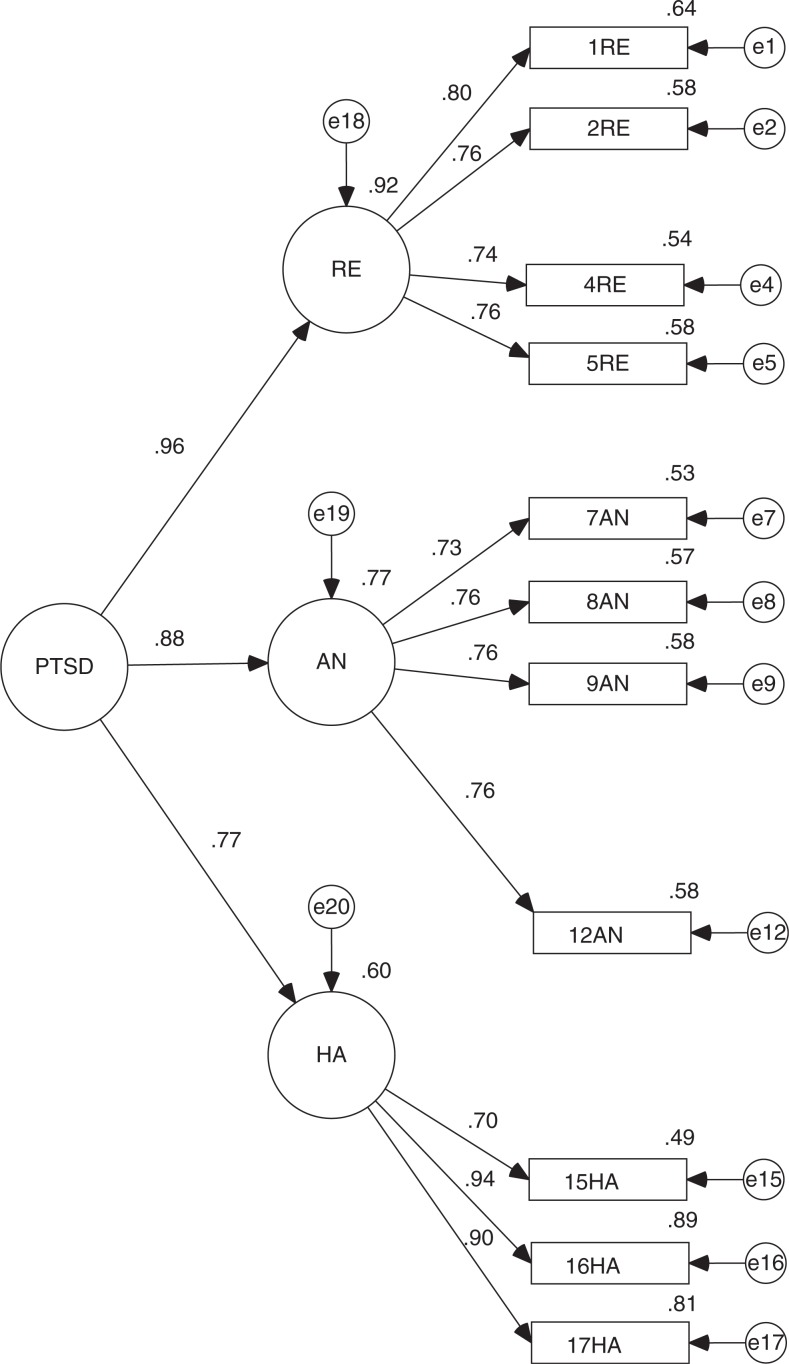
Standardized version of CFA for new DTS-SF second order model.

Despite some fluctuations in its fits, confirming the validity of DTS in three samples obtained after a single shocking and potentially traumatic event (F-27) and the creation of a newly reduced version of the same scale (DTS-SF) produced two important findings from this investigation. We confirmed the structure found by other authors based on three factors (Bobes et al., 2000; Chen et al., 2001; Seo et al., 2008; Villafañe et al., 2003) for the original version and that of 11 items. Furthermore, given the important correlations between latent variables or scale dimensions, it is possible to establish a second-order variable that we could call PTSD (Figs. 4 and 5). Both models show the same fit indicators as its predecessors and its existence reinforces the validity of the scale in both its versions. We could say, then, that both DTS and DTS-SF are valid scales for measuring PTSD.

Our attention is called to the fact that DTS-SF shows somewhat better indicators than the original version. Although there is another short version called SPAN (named for its four items Startle, Physiological arousal, Anger and Numbness; Meltzer-Brody, Churchill, & Davidson, 1999), we did not find any validation study with EFA and/or CFA (Chen, Shen, Tan, Chou, & Lu, 2003; Seo et al., 2011; Yeager, Magruder, Knapp, Nicholas, & Frueh, 2007), which makes us doubt its construct validity. This represents an opportunity, as it is possible to apply a shorter version of DTS as valid as the original and with the same factor structure. Therefore, with DTS-SF, we would have more certainty of measuring the same construct. DTS-SF could be very useful in situations where participants present a higher degree of emotional stress, a lower degree of reading comprehension, or in situations in which the application of considerably long instruments is difficult. In addition, in order to confirm the validity of this version, we propose performing other investigations that compare the functioning of DTS-SF with other PTSD measures serving as criteria (i.e., PCL-C, TOP-8, SPRINT-E; Bobes et al., 2000; Norris et al., 2008; Leiva-Bianchi & Gallardo, 2013; Weathers, Litz, Herman, Huska, & Keane, 1993). Certainly, we recommend testing the construct validity of SPAN and to compare it with DTS and DTS-SF validity.

In regard to criterion measures, we used only one in our investigation: the degree of damage to the home. The relationship between DTS and damage to the home, despite being scarce (63%), weak (*r*<0.3) and insignificant, is useful in order to confirm scale validity. As we know, the probability of suffering from PTSD increases with the person's exposure to the event, whether by heavy damage to their belongings after a catastrophe (MIDEPLAN, 2011) or for other socio-demographic and environmental factors (Goenjian et al., 2000). Nevertheless, it is important to note that this is not a casual relationship, which is to say that not all those who suffered a high degree of exposure to the event will necessarily suffer from a psychopathology such as PTSD. In fact, the majority spontaneously recovers, and even experiences personal growth after the event (Tedeschi & Calhoun, 2004).

Here, we can mention the study's first limitation. There are probably other more precise criterion measures than the one used in this case. In fact, the PTSD and post-disaster stress scales mentioned are a good alternative for this purpose. Unfortunately, the measurements carried out in the CASEN Survey did not consider these types of measurements as criteria. However, the third sample used in this study did consider two other criterion measures: the SPRINT-E and a checklist to determine the presence or absence of panic attack symptoms. Complementarily, we would like to note that upon making Pearson correlations between the 17 DTS items and the SPRINT-E scale totals and the panic attack symptoms, all items proved to be significantly correlated with both criteria (*p*<0.01).

Another limitation might be the maximum likelihood as a method of extraction. While this method is widely used in CFA and provides statistical tests to estimate model parameters (Martinez, Hernandez, & Hernandez, 2006), it may not be the most appropriate when variables are ordinal or when they do not meet normality assumption (Brown, 2006; McIntosh, 2007), such as the DTS items (see Table 3). Therefore, we recommend performing the analysis with another method, such as unweighted least square (ULS; Brown, 2006). Complementary to presented results and based on the sample three, we performed the analysis of DTS and DTS-SF by ULS. This procedure brings us to confirm the results for DTS (RMR =0.252, GFI = 0.990, NFI = 0.988 and PNFI = 0.843) and DTS-SF (RMR = 0.177, GFI = 0.996, NFI = 0.994, and PNFI =0.745). Performing some items transformation procedure could have been another solution (e.g., log or square root). However, this could hinder the interpretation of results in having to change from original to transformed scores.

**Table 3:** Descriptive statistics for DTS items

	Sample 1			Sample 2			Sample 3		
			
Item	M	SD	S	M	SD	S	M	SD	S
1RE	1.145	1.763	1.771	1.295	1.815	1.448	2.160	2.195	0.952
2RE	0.830	1.563	2.128	0.820	1.424	1.946	1.365	1.980	1.518
3RE	0.600	1.345	2.188	0.600	1.272	2.252	1.555	2.017	1.215
4RE	0.810	1.512	1.993	0.945	1.648	1.606	2.640	2.113	0.825
5RE	0.620	1.402	2.437	0.525	1.307	2.626	1.350	2.133	1.618
6AN	0.500	1.260	2.747	0.485	1.195	2.868	1.605	2.175	1.378
7AN	0.305	0.973	3.322	0.485	1.228	2.742	1.355	2.145	1.684
8AN	0.615	1.482	2.564	0.245	0.865	4.580	1.300	1.921	1.664
9AN	0.395	1.203	3.402	0.580	1.335	2.649	1.275	1.990	1.829
10AN	0.315	0.990	3.723	0.210	0.793	4.612	1.065	1.791	1.954
11AN	0.600	1.467	2.640	0.180	0.768	5.585	1.140	1.892	1.790
12AN	0.860	1.663	1.875	0.365	1.090	3.562	1.630	2.289	1.418
13HA	0.595	1.338	2.414	0.845	1.601	1.903	2.135	2.587	0.987
14HA	0.730	1.417	2.073	0.555	1.366	2.685	1.835	2.257	1.063
15HA	1.005	1.806	1.776	0.515	1.264	2.710	2.055	2.275	0.968
16HA	1.170	1.936	1.540	0.905	1.590	1.846	2.220	2.435	0.863
17HA	0.635	1.401	2.325	1.000	1.653	1.617	2.430	2.509	0.773

Note: All items are not normally distributed according to the Kolmogorov–Smirnov and Shapiro–Wilk (*p*<0.05).

Although it is not the first DTS validation in Spanish, it is the first time in which two samples have been taken via a random sampling procedure. This strength is not common in DTS validation studies reviewed, or in any other validation study that we know of, for that matter. This strength allows us to arrive at a conclusion in respect to the DTS structure that is most representative of the population affected by the same potentially traumatic event. This, together with the high validity of the scale criteria and the fact that validation was carried out after a single stressful event common to all participants (F-27), allows for a decrease in the margin of error of estimates and assurance of the accuracy of the validation results. In addition, this effort is relevant when having representative cross-cultural findings.

As per the particular characteristics of the 2010 CASEN Survey database, we can mention that further analysis represents at least three interesting psychometric opportunities. The first pertains to performing DTS validation with those participants that received PTSD diagnosis (a total score of 40 points or more on the scale) and not with the general population, as was the case in this study. The second consists of dividing the total sample number (26,737) into 138 samples of 200 participants each, validating each sample and finally obtaining an indicator of average or proportion fit for DTS. The third opportunity is related to performing analysis using the item response theory, given the large number of participants that responded to the DTS as part of the CASEN Survey.

Another task still pending is the creation of norms that confirm (or not) the issue of the 40 points originally provided (Davidson et al., 1997). For this, we propose applying DTS accompanied by a structured clinical interview (the gold standard for validation) in order to generate the ROC curves necessary for establishing its sensitivity. The emphasis here on the search for a better model for DTS was detrimental to these important aspects. Nevertheless, in order to partially mitigate this weakness and provide practical criteria for decision making in the clinical environment, we used the 40-point limit mentioned for calculating PTSD prevalence in the population. We found that for the three samples analyzed, there would be 10, 6, and 28% prevalence of PTSD, respectively, that is, an average of 15%. This concurs with the prevalence results mentioned at the beginning. However, given the variation of PTSD prevalence in the three samples, we suggest further investigation of factor structures across groups with high, mid and low levels of symptoms.

For its part, the new 11-item version does not possess scales either. However, we note as a reference that the equivalent of the 40 points on the original scale correspond to the scores of 28, 30, and 27 for the three samples on the scale of 11 points applied here. Therefore, scores above 28 points on DTS-SF imply a higher risk of the presence of PTSD. Finally, we recommend incorporating DSM-V criteria for diagnosing PTSD in future evaluations. In this regard, it is important to include assessments of negative moods and dissociated thoughts. To differentiate the symptoms of PTSD with panic attacks could be relevant in order to identify cases of PTSD (Friedman, Resick, Bryant, & Brewin, 2011). Studies of comorbidity and differential diagnosis of disorders based on fear and anxiety (e.g., panic attacks), depression, and dissociative disorders would be very useful in this regard.

## Author's note

The submitted article has not been previously published or orally presented, and is not under consideration elsewhere.

The corresponding author is not a member of ESTSS.
